# Effect of three edible oils on the intestinal absorption of caffeic acid: An *in vivo* and *in vitro* study

**DOI:** 10.1371/journal.pone.0179292

**Published:** 2017-06-15

**Authors:** W. Chaturi Prasadani, Chaturi M. Senanayake, Nimanthi Jayathilaka, Sagarika Ekanayake, Kapila N. Seneviratne

**Affiliations:** 1 Department of Chemistry, Faculty of Science, University of Kelaniya, Kelaniya, Sri Lanka; 2 Department of Biochemistry, Faculty of Medical Sciences, University of Sri Jayawardenapura, Nugegoda, Sri Lanka; University of Palermo, ITALY

## Abstract

Polyphenolic antioxidants are mainly absorbed through passive paracellular permeation regulated by tight junctions. Some fatty acids are known to modulate tight junctions. Fatty acids resulting from the digestion of edible oils may improve the absorption of polyphenolic antioxidants. Therefore, we explored the effect of three edible oils on the intestinal absorption of caffeic acid. Rats were fed with soybean oil and caffeic acid dissolved in distilled water. Caffeic acid contents in the plasma collected up to 1 hr were quantified. The experiment was repeated with coconut oil and olive oil. Component fatty acids of the oils were individually tested *in vitro* for their effect on permeability of caffeic acid using Caco-2 cell monolayers. Highest absorption of caffeic acid was observed in animals fed with coconut oil. *In vitro* transport percentages of caffeic acid in 2.5 mmol/L solutions of fatty acids were 22.01±0.12 (lauric), 15.30 ± 0.25 (myristic acid), 13.59 ± 0.35 (linoleic acid), 3.70 ± 0.09 (oleic acid) and 0.10–2.0 (all other fatty acids). Lauric acid and myristic acid are the two major fatty acids present in coconut oil. Therefore, these fatty acids may contribute to the higher absorption of caffeic acid in the presence of coconut oil.

## Introduction

Polyphenols are naturally occurring secondary metabolites found in plants. Edible sources of polyphenolic substances are vegetables, fruits, cereals, and beverages such as tea, coffee and wine. Long term consumption of diets rich in plant polyphenolic substances has been reported to confer protection against development of cardiovascular diseases, cancers, diabetes, osteoporosis and neurodegenerative diseases [1–3]. Polyphenols can also inhibit cholesterol uptake and 5-lipoxygenase activity [4]. Bioavailability of phenolic substances is important in evaluating the health benefits of phenolic substances. Bioavailability of a substance indicates the fraction of an ingested nutrient or compound that reaches the systemic circulation and the specific sites where it can exert its biological action [5]. Even though a compound has strong antioxidative or other biological activities *in vitro*, *in vivo* biological activity depends on the ability of the compound to reach the target tissue [6]. An absorption study conducted with human subjects indicate that at least 55–66% of the ingested dose of olive oil phenolic substances are absorbed by humans [7]. However, absorption of dietary polyphenols in the small intestine has been reported to be low (10% to 20%) [8].

Passive paracellular absorption, regulated by tight junctions is the main route for absorption of poorly absorbed hydrophilic substances such as polyphenolic antioxidants. Tight junctions act as a paracellular barrier towards permeation of small polar molecules that are water soluble [9]. Impairment of the tight junction function is of importance in pathogenesis of various diseases [10]. However, tight junction modulation by some compounds without causing intestinal inflammation may be beneficial, as it allows the passage of poorly absorbing polar molecules. For example, luminal sodium caprate, a food constituent, can increase tight junction permeability, allowing passage of macromolecules, without affecting epithelial viability [11]. Use of sodium caprate as an enhancer of drug absorption has been well-documented [12]. Capric acid (C10), lauric acid (C12) and oleic acid (C18) significantly increase levothyroxine sodium transport and the order of enhancement was C12≈C18 > C10. This increase in transport and the reductions in transepithelial electrical resistance (TEER) values indicate opening of tight junctions to improve the paracellular permeability [13]. Sodium caprate induced increased permeability to polysucrose and opening of the tight junctions was visualized by transmission electron microscopy [11]. Effect of polyunsaturated fatty acids on tight junction permeability has been investigated and the results indicate that γ-linolenic acid, α-linolenic acid, and eicosapentaenoic acid significantly increased TEER whereas linoleic acid significantly reduced TEER of ECV304 cells [14]. Capric acid, lauric acid and conjugated linoleic acid (CLA) have been shown to improve paracellular calcium transport across Caco-2 cells [15]. The distribution of integral structural components of the tight junctions, Occludin and ZO-1 peptides, which are involved in the biogenesis and functional integrity of the epithelial monolayer, was altered by *trans*-10 CLA isomer indicating that this fatty acid affects the permeability [16]. During the course of the absorption, polyphenols undergo extensive modification by conjugating to glucuronide, sulfate and methyl groups in the gut mucosa and inner tissues [17]. Many diets contain edible oils and polyphenolic substances. Herein, we report the effect of three common edible oils on the absorption of caffeic acid using a rat model. The effect of major fatty acids in the three tested oils on the absorption of caffeic acid was also tested using Caco-2 cell monolayers to support the findings of the animal study.

## Materials and methods

### Determination of fatty acid composition of different oils

The fatty acid composition of phenol-stripped oils used in the present study was analyzed by the method given by Seneviratne and Kotuwegedara without any modifications [18].

### Animal study

Male weaning Wistar rats weighing 210–230 g were randomly collected from the Medical Research Institute, Colombo. They were placed individually in cages and housed in a room with a temperature range of 25 ± 2°C with a 12 h light-darkness cycle. Prior to the initiation of the experiment, the rats were acclimatized to the basal diet for two weeks. Then the rats were randomly assigned to treatment groups (6 rats/group). The basal diet was replaced with a diet prepared without adding any source of fat, 16 hrs prior to the oral feeding experiments.

Rats were orally administered phenol-stripped soybean oil (1 mL) and 700 μmol/kg caffeic acid dissolved in distilled water (2 mL). The second group of rats was given phenol-stripped coconut oil (1 mL) and 700 μmol/kg caffeic acid dissolved in distilled water (2 mL) and the third group was given phenol-stripped olive oil (1mL) and 700 μmol/ kg caffeic acid in water (2 mL). The rats were anaesthetized and blood was collected into EDTA tubes through the tail vein, 30 min after oral administration. The blood samples were centrifuged promptly (3000 g, 10 min) at room temperature. The top layer of plasma was stored at -20°C for further analysis. After drawing blood, the basal diet was provided to the animals. Two days after first drawing of blood, the basal diet was replaced with a diet prepared without adding any source of fat prior to oral feeding experiments as described above. Rats were orally administered phenol-stripped soybean oil (1 mL) and 700 μmol/kg caffeic acid dissolved in distilled water (2 mL) and blood was collected 45 min after oral feeding and the same experiment was repeated for second and third groups of rats with coconut oil and olive oil respectively. This feeding cycle was carried out with the three oils for collection of blood after 60 min. Phenol-stripped oils were prepared according to a reported method [19]. This study was carried out in strict accordance with the recommendations of the Ethics Review Committee of University of Sri Jayawardenapura, Sri Lanka. The experimental protocol was approved by the ethics review committee of University of Sri Jayawardenapura, Sri Lanka. The same ethics review committee specifically reviewed and approved the use of ether in the study design (Permission Number -13/14). The rats were anaesthetized humanely by exposing them to anesthetizing ether for a short period of time for sample collection to minimize suffering. At the end of the study period, animals were sacrificed by exposing to anesthetizing ether according to the procedure that was approved by the Animal House of Medical Research Institute, Sri Lanka. Use of mild anesthetizing ether does not affect the *in vivo* antioxidant properties according to our previous studies [20]. This study only investigated the absorption of caffeic acid compared to control group without fatty acid treatment and the antioxidant capacity therein. Therefore, the experimental procedure was reviewed and approved by the above mentioned ethics review committee.

#### Analysis of plasma caffeic acid

Caffeic acid content was determined by high-performance liquid chromatography (HPLC). Plasma (200 μL) was added to sodium acetate buffer (50 μL, pH 5.0, 0.1 mol/L) and methanol (250 μL). The mixture was vortexed at 40 Hz for 30 s, sonicated at 40 kHz for 10 min at RT, and vortexed again at 40 Hz for 30 s and was centrifuged (5000 g, 5 min). Elagic acid was used as the internal standard (IS) and the supernatant (20 μL) was injected in to the chromatograph. HPLC experiments were performed using an Agilent 1100-Infinity liquid chromatographic system (Agilent Technologies, Waldbronn, Germany) equipped with an Agilent 1200 diode array detector. A ZORBAX ECLIPSE Plus C18 column (Agilent Technologies, USA) (4.6 x 100 mm x 3.5 μm particle size) maintained at RT was used for this purpose. The mobile phase consisted of 1x10^-3^ mol/L H_2_SO_4_ in deionised water (A) and methanol (B). The total running time was 20 min with a flow rate of 0.5 mL/min. The elution gradient began using 90% A/10% B and the solvent composition was changed to 45% A and 55% B, at 10 min. This composition (45% A/55% B) was continued for 10–20 min. Caffeic acid was detected at 280 nm.

#### Enzymatic hydrolysis of caffeic acid conjugates and determination of the caffeic acid concentration in plasma

Plasma (50 μL) was mixed with sulfatase/*β*-glucuronidase solution (50 μL) in sodium acetate buffer (0.1 mol/L, pH 5.0). The mixture contained 500 units of *β*-glucuronidase and 25 units of sulfatase enzyme. The mixture was incubated at 37°C for 4 h. Released compounds were extracted and analyzed by HPLC as described in 2.3. The resultant amount of caffeic acid after the enzyme treatment was considered to be proportional to the amount of caffeic acid absorbed. Caffeic acid in all experiments was quantified using a standard curve as previously described [21].

#### Ferric reducing antioxidant power (FRAP) assay

The reducing power of the plasma was determined using FRAP assay [22]. FRAP working solution was prepared by mixing acetate buffer (300 mmol/L, pH 3.6), 2,4,6-tripyridyl-s-triazine (TPTZ) (10 mmol/L in 40 mmol/L HCl) and FeCl_3_.6H_2_O (20 mmol/L) at a 10:1:1 ratio. FRAP working solution (280 μL) was mixed with plasma (20 μL). After 4 minutes of incubation at 37°C, the absorbance was measured at 593 nm using MultiSkan Go UV-Visible spectrophotometer (Thermo Scientific, Finland) with respect to a blank with no added caffeic acid.

### Cell culture

Caco-2 cells [23] were cultured according to a previously published method [24]. For the transport measurements, cells were seeded in transwells at 1 x 10^5^/cm^2^ and allowed to grow for 21 days. The medium Dulbecco’s MEM (DMEM) containing 10% fetal bovine serum was changed every other day. After 21 days, TEER values of the cells were measured using Millicell-ERS equipment (Millipore, MA). Wells with TEER > 260 Ω.cm^2^ were used for the transport studies and the TEER values of the monolayer was measured before and after transport experiment. The medium was removed from the apical and basal sides of the monolayer and 2 mL of sample solution was introduced to the apical side and 2 mL of 1.8 mmol/L Ca^2+^ in Hank’s balanced salt solution (HBSS) was introduced to the basal side. For the control experiment, 2 mL of control solution was introduced to the apical side and 2 mL of 1.8 mmol/L Ca^2+^ in Hank’s balanced salt solution (HBSS) was introduced to the basal side. After 1 h, 1.00 mL of basal solution from each well was collected into an eppendorf tube containing 25 μL of 4 mol/L acetic acid. Contents in the eppendorf tubes were mixed and centrifuged (5000 g, 10 min). Then the top solution was collected and analyzed by HPLC as described above for caffeic acid. The transport percentages were calculated by comparing the concentration of caffeic acid in the solutions before and after the transport experiments using HPLC signal areas.

Sample solution was prepared as follows: Caffeic acid (2.20 mg) and Lucifer yellow (2.50 mg) were dissolved in 25 mL of calcium free HBSS in a volumetric flask. Two 10.00 mL aliquots of this solution were transferred to two 50 mL centrifuge tubes. Tween 80 (5 μL) emulsifier was added to each tube. To one tube, fatty acid was added to give a 2.5 mmol/L concentration. The mixtures in the tubes were homogenized at 37°C and the pH was adjusted to 7.4 by 10 mol/L HCl or 10 mol/L NaOH. The solution with no added fatty acid was considered as the control solution.

#### MTT (3-(4,5-dimethylthiazol-2-yl)-2,5-diphenyl tetrazolium bromide) assay

MTT solution was prepared by dissolving 15.0 mg of MTT in 30 mL of serum free medium. After the transport experiment, cells were washed twice with serum free medium (2 mL/transwell each time). MTT solution (2 mL) was added to the apical chamber and incubated at 37°C for 4 h. Then the solution was removed from the apical and basal chambers and the blue crystals were dissolved in acid-isopropanol as described by Mosmann [25]. A portion of 300 μL from each solution was transferred to a plate and the absorbance was read at 570 nm. Percentage viability was calculated according to the following formula:

$$\text{Viability }\left(\%\right)\;=\;({\text{I}}_{\text{s}}/{\text{I}}_{\text{c}})\text{ x }100$$

(I_s_ = Color intensity of the sample, I_c_ = Color intensity of the control with no added fatty acid)

### Data analysis

Cell culture experiments were run in triplicate. Data in animal studies are presented as means ± SD with n = 6. The student's t-test was adopted at a significance level of p<0.05 to determine statistically significant differences among the experimental groups.

## Results and discussion

### Fatty acid composition

Plant-based oils contain minor polar components such as phenolic antioxidants, tocopherols, free fatty acids, mono- and diacylglycerols and phospholipids. Phenol-stripping process removes all these minor components [26]. Quantities of major (above 5%) fatty acids of phenol-stripped oils are as follows: soybean oil—linoleic acid (55.20 ± 1.76), oleic acid (23.88 ± 1.15) and palmitic acid (14.68 ± 0.32); coconut oil—lauric acid (51.93 ± 3.31), myristic acid (19.30 ± 0.76), caprylic acid (8.64 ± 0.23), and capric acid (6.42 ± 0.21); olive oil—oleic acid (71.62 ± 1.78), palmitic acid (15.11 ± 1.61) and linoleic acid (6.19 ± 0.50). These three oils were selected because of their unique composition of different fatty acids. Soybean oil was selected as the linoleic acid oil. Coconut oil was selected as the short and medium chain fatty acid oil while olive was selected as the monounsaturated fatty acid oil.

### Animal study

**Fig 1:**
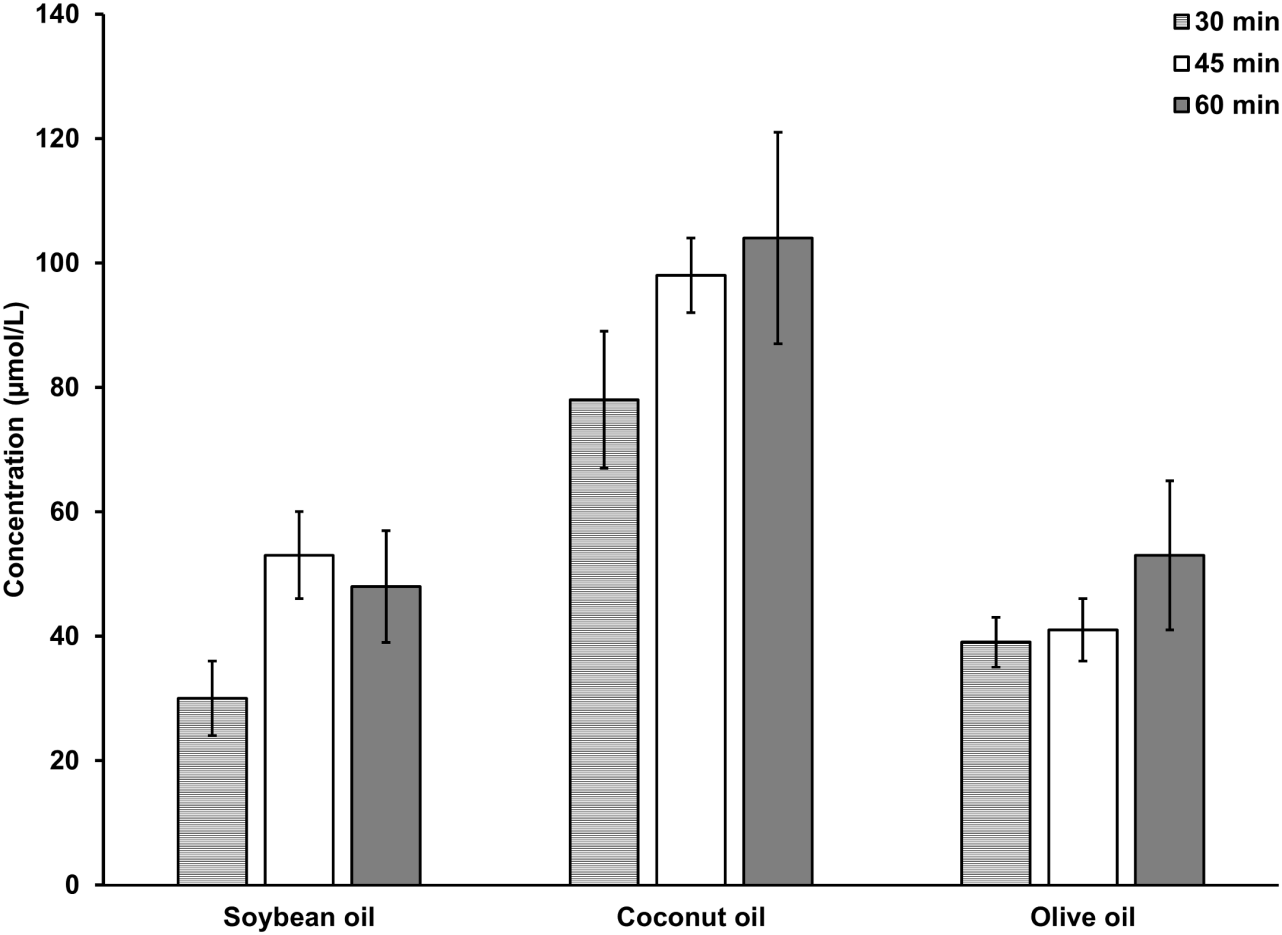
Concentration (μmol/L) of caffeic acid in plasma collected after oral feeding and hydrolyzed with sulfatase / *β*-glucuronidase. Concentration of total caffeic acid in Soybean oil treated group and the Olive oil treated group were significantly lower compared to the Coconut oil treated group up to 1 hr after treatment (p<0.05). n = 6.

The study was conducted to investigate the effect of the three selected edible oils on the absorption of caffeic acid, which represents a small polar phenolic antioxidant molecule. Oils are digested in the intestine in to fatty acids and it was hypothesized that the component fatty acids of the three oils have different effects on the absorption of polar molecules. Transport of caffeic acid is linear within 30–45 min period after oral feeding for soybean oil and coconut oil according to Fig 1. After 45 min, a linear increase of caffeic acid content was not clearly observed in plasma. Phenolic antioxidants form metabolic products during absorption, which facilitates the excretion of these compounds [27]. However, during the 30–45 min period after feeding, either free caffeic acid in plasma or the caffeic acid content after treating plasma with de-conjugating enzymes can be used to study the effect of oils on the absorption of caffeic acid. Three hrs after feeding, free caffeic acid was not observed in the plasma and the ferric reducing antioxidant power of plasma was equal to the controls with no added caffeic acid.

**Fig 2:**
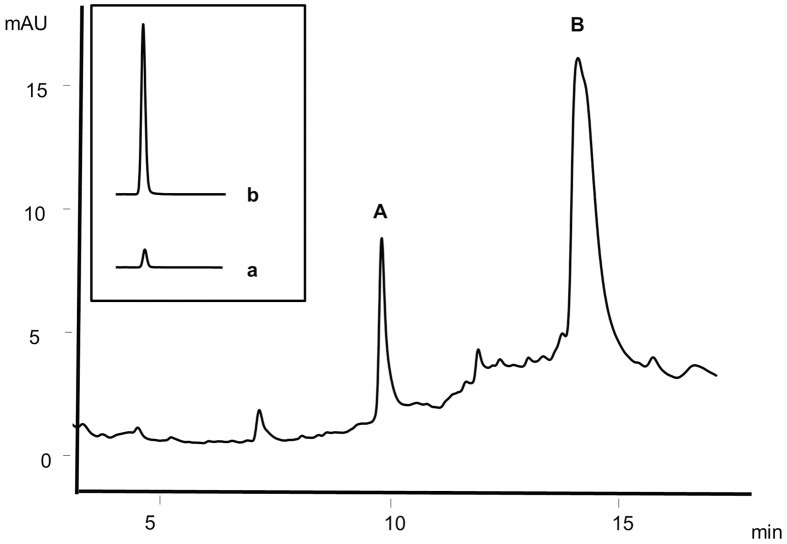
HPLC chromatogram of plasma after treating with de-conjugating enzymes. A, caffeic acid and B, elagic acid (Internal standard). Inset: Signal areas of caffeic acid transported through Caco-2 cell monolayers; a, caffeic acid signal in the control with no added lauric acid, b, caffeic acid signal in the sample with added lauric acid at 2.5 mmol/L.

Absorbed caffeic acid content in the plasma during 30–45 min after oral feeding of rats was used in the present study to evaluate the effect of fatty acids on the permeability of caffeic acid by treating the plasma with de-conjugating enzymes. The results indicate that the rats fed with coconut oil show the highest levels of caffeic acid in plasma and the amount of caffeic acid absorbed for coconut oil is significantly higher (p<0.05) than that for soybean oil and olive oil up to 1 hr as shown in Fig 1. The HPLC chromatogram of the de-conjugating enzyme-treated plasma is given in the Fig 2. Caffeic acid was identified by comparison of retention time as well as by spiking the plasma with an authentic standard of caffeic acid.

**Fig 3:**
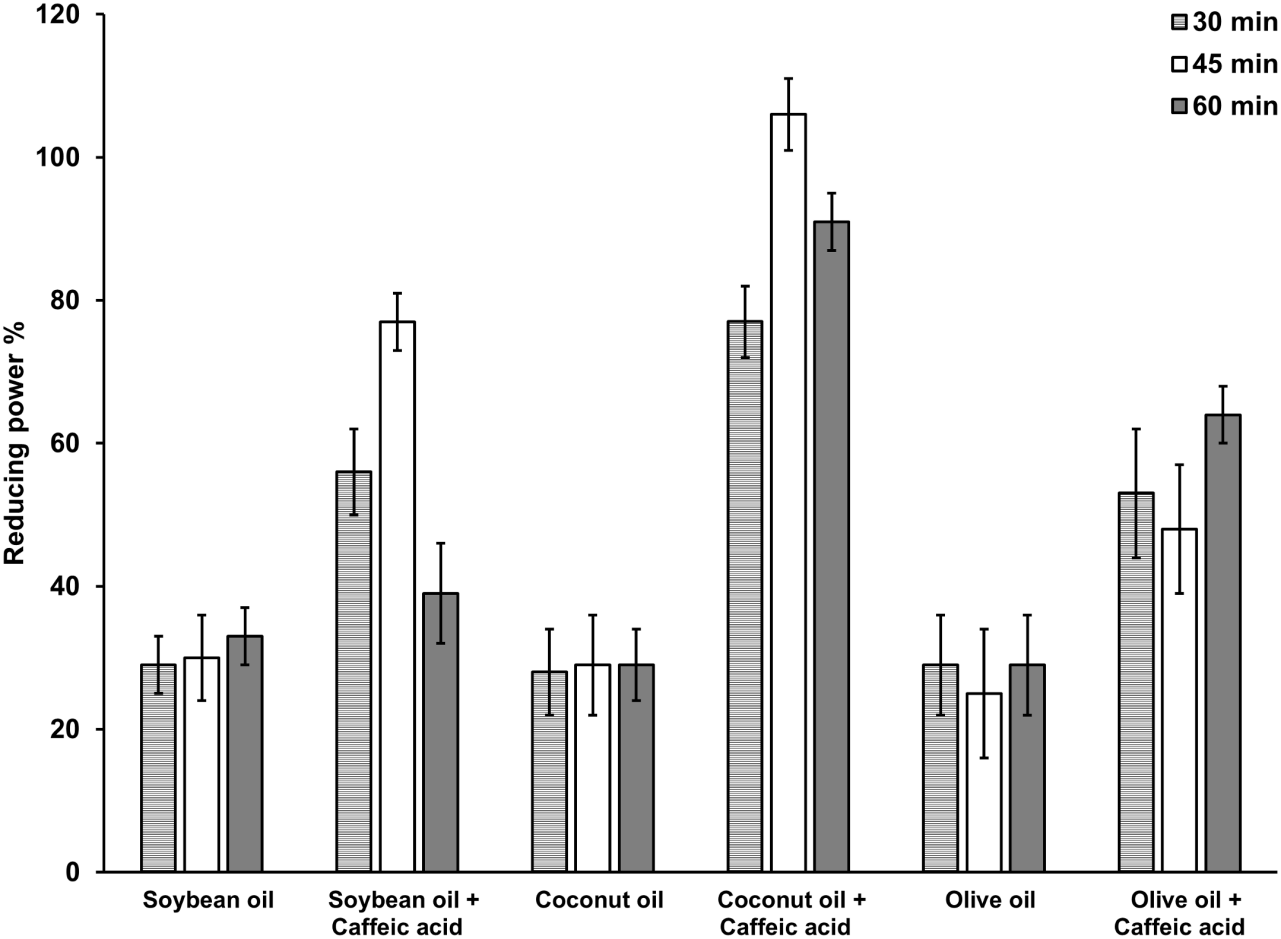
Ferric reducing antioxidant power (reducing power %) of plasma. Ferric reducing antioxidant power of Soybean oil and caffeic acid treated group and the Olive oil and caffeic acid treated group were significantly lower compared to the Coconut oil and caffeic acid treated group up to 1 hr after treatment (p<0.05). n = 6.

Polyphenols, when consumed are known to improve antioxidant capacity in blood. Green tea beverage and green tea extracts significantly increased serum antioxidant capacity due to high polyphenol contents in human subjects [28]. FRAP assay has been used previously to evaluate plasma antioxidant capacity in volunteers who consumed red wine, which is rich in polyphenols [29]. In the present study, antioxidant capacity of plasma evaluated by FRAP assay indicates that there is a significant increase of serum antioxidant capacity in the animals fed with caffeic acid in the presence of all three oils compared to control groups fed with caffeic acid without oils (Fig 3). The antioxidant capacity of plasma of rats fed with coconut is significantly higher (p<0.05) compared to that of plasma of rats fed with soybean oil or olive oil correlating with the higher absorption of caffeic acid in the presence of coconut oil.

### Cell culture

As given in the introduction, C10, C12 and C18 fatty acids are known to promote the absorption of polar molecules through tight junctions. Though individual fatty acids have been tested *in vitro* for their effect on improving the permeability of various compounds, this is the first report of the effect of edible oils on the absorption of phenolic antioxidants using an animal model followed by the confirmation of the results of animal study by *in vitro* cell permeability assay. Permeability of caffeic acid with all the major fatty acids were individually tested using Caco-2 cell monolayers. The free caffeic acid contents transported through the Caco-2 cell monolayers were, calculated using the signal areas of free caffeic acid in the HPLC chromatograms. The signal areas of the chromatograms of caffeic acid in the basal solution after transport of caffeic acid solutions with and without lauric acid are given in Fig 2b and 2a respectively (inset) as representative signals. No metabolic products of caffeic acid were detected in significant amounts up to 1 hr of transport. In previous studies, ferulic acid was efficiently transported as the free form through an *in vitro* model for the colonic epithelium consisting of cocultured Caco-2 and mucus-producing HT29-MTX cells, with no considerable metabolism [30].

It is not possible for the substances that can pass only through tight junctions to cross cell monolayer when tight junctions are well maintained [31]. In the present study, the improvement of tight junction permeability by fatty acids was further demonstrated with lucifer yellow, which travels across cell monolayer only by paracellular diffusion through tight junctions. The integrity of the monolayer was measured by monitoring the rejection of lucifer yellow, a marker for paracellular diffusion across the cell monolayer. The transport percentages of the fatty acids and lucifer yellow are given in the Table 1. Free caffeic acid content in the transported solutions containing fatty acids were significantly higher than the free caffeic acid contents in the transported solutions with no added fatty acids (p<0.05). However, a remarkably noteworthy increase of permeability of caffeic acid was observed for lauric acid, myristic acid and linoleic acid compared to other fatty acids. A similar trend of permeability can be seen for lucifer yellow as well in the presence of these three fatty acids. Cell viability for all the tested fatty acids after 1 h was above 96%. Transport studies were not successful with oils instead of fatty acids at any concentration due to poor cell viability reflected by MTT assay.

**Table 1:** Transport percentages of caffeic acid and lucifer yellow after 1 hr of transport through a Caco-2 cell monolayer.

Fatty acid	Caffeic acid transported (%)		Lucifer yellow transported (%)	
	Test solution	Control	Test solution	Control
Caprylic (C8)	1.10 ± 0.03	0.60 ± 0.01	0.35 ± 0.01	0.11 ± 0.01
Capric (C10)	2.01 ± 0.10	0.71 ± 0.04	0.76 ± 0.02	0.45 ± 0.01
Lauric (C12)	22.01 ± 0.12	1.08 ± 0.07	21.13 ± 0.40	0.25 ± 0.03
Myristic (C14)	15.30 ± 0.25	1.00 ± 0.13	9.76 ± 0.37	0.45 ± 0.01
Palmitic (C16)	1.10 ± 0.13	0.70 ± 0.04	0.40 ± 0.03	0.21 ± 0.01
Oleic (C18:1)	3.70 ± 0.09	0.70 ± 0.04	3.45 ± 0.18	0.27 ± 0.01
Linoleic (C18:2)	13.59 ± 0.35	1.08 ± 0.09	11.26 ± 0.10	0.51 ± 0.01

Absorption of caffeic acid and Lucifer yellow significantly enhanced with all fatty acids under investigation compared to controls (p<0.05). n = 3

The absorption experiments conducted with rats indicate that highest absorption of caffeic acid is evident in the presence of coconut oil. Lauric acid, myristic acid and linoleic acid, show the highest impact on absorption of caffeic acid in transport studies using Caco-2 cell monolayers. Capric acid, lauric acid and oleic acid are known to increase the permeability of polar drugs in other cell monolayers [13]. Lauric acid, myristic acid and capric acid are among the major fatty acids present in coconut oil. Therefore, the highest absorption of caffeic acid in the animals in the presence of coconut oil may be attributed to the fatty acid composition of coconut oil. Absorption efficiencies of caffeic acid in the presence of soybean oil and olive oil in the animal study can also be explained by the *in vitro* transport efficiencies of caffeic acid through cell monolayers in the presence of major component fatty acids of these two oils. However, strict empirical relations between the improvement of absorption of caffeic acid by oils and the fatty acid composition of oils cannot be drawn based on the results of the present study. Tight junctions are composed of transmembrane proteins such as occludin and claudins. Phosphorylation of tight junction proteins can disrupt the tight junctions [32]. There are indications of the effect of *trans*-10 CLA and some metabolites of fatty acids on altering the tight junctions and barrier function [33]. Such studies for all the major fatty acids in edible oils will be helpful in predicting the effect of edible oils on the absorption of small polar molecules through paracellular pathway in the intestine. Ultimate nutritional aspects of phenolic antioxidants and effectiveness of drugs depend on their bioavailability. Findings of the present study suggest that lauric acid, myristic acid, linoleic acid and oleic acid or the oils containing mainly these fatty acids may improve the bioavailability of polar antioxidant molecules, thereby improving their nutritional properties. Therefore, coconut oil may have the highest impact on bioavailability of polar antioxidant molecules.

## Supporting information

S1 Table Concentration of caffeic acid in plasma collected after oral feeding and hydrolyzed with sulfatase / *β*-glucuronidase. (DOCX)

S2 Table Ferric reducing antioxidant power (reducing power %) of plasma. (DOCX)

## References

[1] Pandey KB, Rizvi SI. Plant polyphenols as dietary antioxidants in human health and disease. *Oxid Med Cell Longev*. 2009; 2:270–278. doi: 10.4161/oxim.2.5.9498 20716914 10.4161/oxim.2.5.9498 PMC2835915

[2] Crozier A, Jaganath I, Clifford M. Dietary phenolics: chemistry, bioavailability and effects on health. *Nat Prod Rep*. 2009; 26:1001–1043. 19636448 10.1039/b802662a

[3] Noratto G, Porter W, Byrne D, Cisneros-Zevallos L. Identifying peach and plum polyphenols with chemopreventive potential against estrogen-independent breast cancer cells. *J Agric Food Chem*. 2009; 57:5219–5226. doi: 10.1021/jf900259m 19530711 10.1021/jf900259m

[4] Leifert WR, Abeywardena MY. Grape seed and red wine polyphenol extracts inhibit cellular cholesterol uptake, cell proliferation, and 5-lipoxygenase activity. *Nutr Res*. 2008; 28:842–850. 19083497 10.1016/j.nutres.2008.09.001

[5] Porrini M, Riso P. Factors influencing the bioavailability of antioxidants in foods: A critical appraisal. *Nutr*. *Metab*. *Cardiovasc Dis*. 2008; 18:647–650. 18996686 10.1016/j.numecd.2008.08.004

[6] D’Archivio M, Filesi C, Varì R, Scazzocchio B, Masella R. Bioavailability of the polyphenols: status and controversies. *Int J Mol Sci*. 2010; 11:1321–1342. 20480022 10.3390/ijms11041321 PMC2871118

[7] Vissers MN, Zock PL, Roodenburg AJC, Leenan R, Katan MB. Olive oil phenols are absorbed in humans. *J Nutr*. 2002; 132:409–417. 11880564 10.1093/jn/132.3.409

[8] Spencer JPE. Metabolism of tea flavonoids in gastrointestinal tract. *J Nutr*. 2003; 133: 3255S–3261S. 14519823 10.1093/jn/133.10.3255S

[9] Turner JR, Madara JL. Physiological regulation of intestinal epithelial tight junctions as a consequence of Na^+^ -coupled nutrient transport. *Gastroenterology*. 1995; 91:391–1396. 10.1016/0016-5085(95)90605-3 7557112

[10] Bjarnarson I, MacPherson A, Hollander D. Intestinal permeability: An overview. *Gastroenterology*. 1995; 108:1566–1581. 7729650 10.1016/0016-5085(95)90708-4

[11] Söderholm JD, Öman H, Blomquist L, Veen J, Lindmark T, Olaison G. Reversible increase in tight junction permeability to macromolecules in rat ileal mucosa *in vitro* by sodium caprate, a constituent of milk fat. *Dig Dis and Sci*. 1998; 43:1547–1552. 9690393 10.1023/a:1018823100761

[12] Maher S, Leonard TW, Jacobsen J, Brayden DJ. Safety and efficacy of sodium caprate in promoting oral drug absorption: from in vitro to the clinic. *Adv Drug Deliv Rev*. 2009; 61:1427–1449. doi: 10.1016/j.addr.2009.09.006 19800376 10.1016/j.addr.2009.09.006

[13] Pabla D, Akhlaghi F, Zia H. Intestinal permeability enhancement of levothyroxine sodium by straight chain fatty acids studied in MDCK epithelial cell line. *Eur J Pharm Sci*. 2010; 40:466–472. doi: 10.1016/j.ejps.2010.05.002 20580671 10.1016/j.ejps.2010.05.002

[14] Jiang WG, Bryce RP, Horribin DF, Mansel RE. Regulation of tight junction permeability and occludin expression by polyunsaturated fatty acids. *Biochem Biophys Res Comm*. 1998; 244:414–420. 9514943 10.1006/bbrc.1998.8288

[15] Jewell C, Cashman KD. The effect of conjugated linoleic acid and medium-chain fatty acids on transepithelial calcium transport in human intestinal-like Caco-2 cells. *Br J Nutr*. 2003; 89:639–647. 12720584 10.1079/BJN2003835

[16] Roche HM. Terres AM. Black IB, Gibney MJ, Kelleher D. Fatty acids and epithelial permeability: effect of conjugated linoleic acid in Caco-2 cells. *Gut*. 2001; 4:797–802. 10.1136/gut.48.6.797 PMC1728326 11358898

[17] Manach C, Scalbert A, Morand C, Rémésy C, Jime´nez L. Polyphenols: food sources and bioavailability. *Am J Clin Nutr*. 2004; 79:727–747. 15113710 10.1093/ajcn/79.5.727

[18] Seneviratne KN, Kotuwegedara RT. *Canarium zeylanicum* seed oil: an edible oil with beneficial qualities. *Int J Food Sci Technol*. 2009; 44:792–798.

[19] Waraho T, Cardenia V, Rodriguez-Estrada M, McClements DJ, Decker EA. Prooxidant mechanisms of free fatty acids in stripped soybean oil-in-water emulsions. *J Agric Food Chem*. 2009; 57:7112–7117. doi: 10.1021/jf901270m 19572645 10.1021/jf901270m

[20] Seneviratne KN, Hapuarachchi CD, Ekanayake S. Comparison of phenolic-dependent antioxidant properties of coconut oil extracted under hot and cold conditions. *Food Chem*. 2009; 114:1444–1449.

[21] Seneviratne KN, Kotuwegedara RT. Antioxidant activities of the phenolic extracts of seed oils and seed hulls of five plant species. *Food Sci Tech Intl*. 2009; 15:419–425.

[22] Benzie IFF, Strain JJ. Ferric reducing/antioxidant power assay: direct measure of total antioxidant activity of biological fluids and modified version of simultaneous measurement of total antioxidant power and ascorbic acid concentration. *Methods Enzymol*. 1999; 299:15–27. 9916193 10.1016/s0076-6879(99)99005-5

[23] Hughes TE, Sasakg WV, Ordovast JM, Forte TM, Lamon-Fava S, Schaefer EJ. A Novel Cell Line (Caco-2) for the Study of Intestinal Lipoprotein Synthesis. *J Biol Chem*. 1987; 262:3762–3767. 3818664

[24] Konishi Y, Kobayshi S, Shimizu M. Tea Polyphenols Inhibit the Transport of Dietary Phenolic Acids Mediated by the Monocarboxylic Acid Transporter (MCT) in Intestinal Caco-2 Cell Monolayers. *J Agric Food Chem*. 2003; 51:7296–7302. doi: 10.1021/jf034894t 14640574 10.1021/jf034894t

[25] Mosmann T. Rapid colorimetric assay for cellular growth and survival: Application to proliferation and cytotoxicity assays. *J Immunol Methods*. 1983; 65:55–63. 6606682 10.1016/0022-1759(83)90303-4

[26] Waraho T, Cardenia V, Nishino Y, Seneviratne KN, Rodriguez-Estrada MT, McClements DJ, et al Antioxidant effects of mono- and diacylglycerols in non-stripped and stripped soybean oil-in-water emulsions. *Food Res Int*. 2012; 48:353–358.

[27] Scalbert A, Morand C, Manach C, Rémésy C. Absorption and metabolism of polyphenols in the gut and impact on health. *Biomed Pharmacotherapy*. 2002; 56:6276–6282. 10.1016/s0753-3322(02)00205-6 12224598

[28] Basu A, Betts NM, Mulugeta A, Tong C, Newman E, Lyons TJ. Green tea supplementation increases glutathione and plasma antioxidant capacity in adults with the metabolic syndrome. *Nutr Res*. 2013; 33:180–187. 23507223 10.1016/j.nutres.2012.12.010 PMC3603270

[29] Duthie GG, Pedersen MW, Gardner PT, Morrice PC, Jenkinson McE, McPhail DB, et al The effect of whisky and wine consumption on total phenol content and antioxidant capacity of plasma from healthy volunteers. *Eur J Clin Nut*. 1998; 52:733–736. 10.1038/sj.ejcn.1600635 9805220

[30] Poquet L, Clifford MN, Williamson G. Transport and Metabolism of Ferulic Acid through the Colonic Epithelium. *Drug Metab Dispos*. 2008; 36:190–197. 17954526 10.1124/dmd.107.017558

[31] Himanshu R, Jakir P, Pradnya H, Suneel P, Rahul S. The impact of permeability enhancers on assessment for monolayer of colon adenocarcinoma cell line (CaCo-2) used in *in vitro* permeability assay. *J Drug Deliv & Therapeutics*. 2013; 3:20–29.

[32] Kim JH, Kim JH, Jun HO, Yu YS, Kim KW. Protective effect of clusterin from oxidative stress-induced apoptosis in human retinal pigment epithelial cells. *Invest Ophthalmol Vis Sci*. 2010; 51:561–566. 19710412 10.1167/iovs.09-3774

[33] Chattopadhyay R, Dyukova E, Singh NK, Ohba M, Mobley JA, Rao GN. Vascular endothelial tight junctions and barrier function are disrupted by 15(*S*)-hydroxyeicosatetraenoic acid partly via protein kinase Cϵ-mediated zona occludens-1 phosphorylation at threonine 770/772. *J Biol Chem*. 2014; 289:3148–3163. 24338688 10.1074/jbc.M113.528190 PMC3916520

