# Supplementary material for: Effect of three edible oils on the intestinal absorption of caffeic acid: An in vivo and in vitro study
Source: PLoS One. 2017 Jun 15;12(6):e0179292. doi: 10.1371/journal.pone.0179292 (PMC5472295; doi:10.1371/journal.pone.0179292)
Supplement: S1 Table — (DOCX) [file pone.0179292.s001.docx]

**S1 Table. Concentration of caffeic acid in plasma collected after oral feeding and hydrolyzed with sulfatase / *β*-glucuronidase**

|  | Concentration of total caffeic acid in (µmol/L) in the plasma | | |
| --- | --- | --- | --- |
| Oil | 30 min | 45 min | 60 min |
| Soybean oil | 30±6 | 53±7 | 48±9 |
| Coconut oil | 78±11 | 98±6 | 104±17 |
| Olive oil | 39±4 | 41±5 | 53±12 |

Concentration of total caffeic acid in Soybean oil treated group and the Olive oil treated group were significantly lower compared to the Coconut oil treatment group up to 1 hr after treatment (p<0.05). n=6
