# Supplementary material for: Effect of three edible oils on the intestinal absorption of caffeic acid: An in vivo and in vitro study
Source: PLoS One. 2017 Jun 15;12(6):e0179292. doi: 10.1371/journal.pone.0179292 (PMC5472295; doi:10.1371/journal.pone.0179292)
Supplement: S2 Table — (DOCX) [file pone.0179292.s002.docx]

**S2 Table. Ferric reducing antioxidant power (Reducing power %) of plasma**

|  | Reducing power (%) | | |
| --- | --- | --- | --- |
| Feed content | 30 min | 45 min | 60 min |
| Soybean oil | 29±4 | 30±6 | 33±4 |
| Coconut oil | 28±6 | 29±4 | 29±7 |
| Olive oil | 29±6 | 25±7 | 29±5 |
| Soybean oil + Caffeic acid | 56±5 | 77±5 | 39±4 |
| Coconut oil + Caffeic acid | 77±7 | 106±9 | 91±7 |
| Olive oil + Caffeic acid | 53±9 | 48±9 | 64±4 |

Ferric reducing antioxidant power of Soybean oil and caffeic acid treated group and the Olive oil and caffeic acid treated group were significantly lower compared to the Coconut oil and caffeic acid treated group up to 1 hr after treatment (p<0.05). n=6
